# The gut microbiome promotes the growth performance of black soldier fly larvae by detoxifying uric acid

**DOI:** 10.1002/imo2.70070

**Published:** 2025-12-24

**Authors:** Xiaowen Ji, Shaojie Zhou, Wenwen Chen, Bin Cao, Jingjing Zhuang, Sheng Zhang, Bingshuo Han, Benping Yang, Shuxiang Zhang, Yanfeng Xue, Jibin Zhang, Yinglao Zhang, Wei Liu

**Affiliations:** ^1^ Anhui Province Key Laboratory of Crop Integrated Pest Management, School of Plant Protection Anhui Agricultural University Hefei China; ^2^ National Key Laboratory of Agricultural Microbiology, College of Life Science and Technology Huazhong Agricultural University Wuhan China; ^3^ Anhui Province Key Laboratory of Resource Insect Biology and Innovative Utilization, School of Life Sciences Anhui Agricultural University Hefei China; ^4^ College of Animal Science and Technology Anhui Agricultural University Hefei China

## Abstract

This study demonstrates the detrimental effects of exogenous uric acid (UA) on the growth of black soldier fly (BSF) larvae, highlighting the role of gut microbiota in UA degradation. We isolated UA‐degrading bacterial strains associated with BSF, including *Enterococcus faecalis* AHAU24. Genomic analysis identified a UA degradation gene cluster in this strain, providing insights into its potential for UA catabolism. Furthermore, mono‐association of germ‐free larvae with *E. faecalis* AHAU24 confirmed that this strain alleviates UA‐induced growth retardation and enhances bioconversion efficiency by degrading dietary UA.

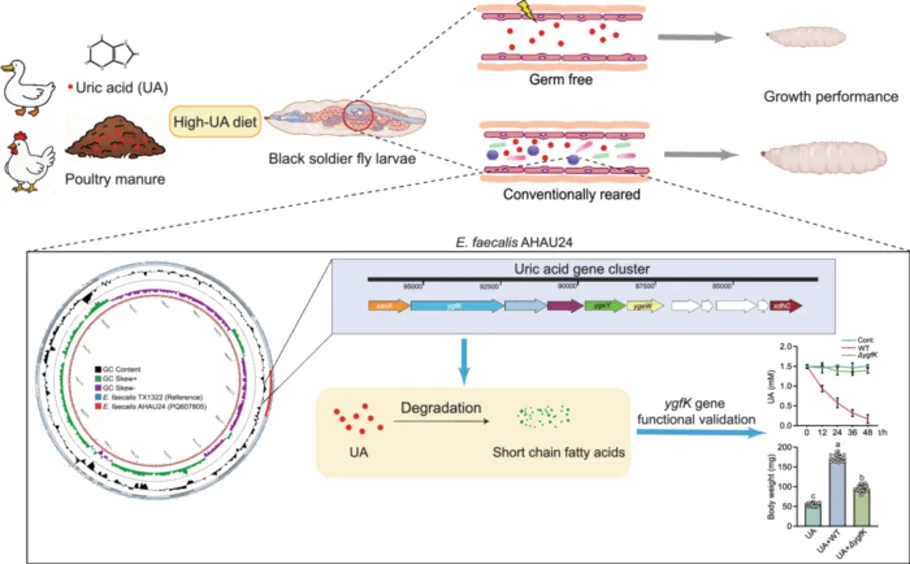

To the editor,

The black soldier fly (BSF, *Hermetia illucens*) has recently garnered increasing attention, due to its remarkable power to convert low‐value organic waste into high‐quality insect biomass [1]. However, many feedstocks contain undesirable or toxic compounds that can adversely affect the growth performance of BSF larvae [2, 3]. For example, poultry manure contains approximately 3% uric acid (UA), which accounts for about 50% of the total nitrogen content in poultry excreta [4]. Notably, it's established that UA is also a major nitrogenous waste product in terrestrial insects [5, 6]. Since UA acts as excreta of both poultry and insects, it is unlikely to be recycled by BSF larvae, raising concerns about its potential toxicity in rearing substrates. Emerging studies reveal that the microbiome, or specific microbial taxa, confer benefits to hosts by degrading a variety of toxins. Certain UA‐related microbes have been recently identified in mammals, in which they contribute to the alleviation of UA‐induced stress [7]. It's therefore proposed that the microbiome could promote the growth performance of BSF larvae by degrading UA. We aim to (I) evaluate the effect of UA on the growth performance of BSF larvae, (II) identify UA‐degrading bacteria associated with larvae, and (III) elucidate the underlying mechanisms by which these bacteria reduce UA toxicity and promote host development.

## 1 BSF LARVAL GROWTH IS NEGATIVELY AFFECTED BY UA STRESS

The conserved conversion of nitrogenous waste to UA facilitates water conservation in terrestrial insects [8]. Consistently, UA accumulated predominantly in the Malpighian tubules of BSF larvae, followed by the cuticle and midgut (Figure 1A), indicating that they follow the paradigm of generating UA as the primary end product of nitrogen metabolism [8]. Next, we investigated whether excess UA could adversely impact the growth performance of BSF larvae. Strikingly, an apparent reduction in body weight and width was observed in larvae exposed to increasing doses of UA (Figure 1B,C). Simultaneously, the larval developmental time was prolonged by increasing UA concentrations (Figure 1D), accompanying a reduction in the pupation rate of larvae (Figure 1E). Altogether, our data suggested that UA negatively affected the growth and development of BSF larvae. Given that BSF larvae are highly efficient decomposers, we finally evaluated whether UA could affect bioconversion rates of food. The bioconversion rate plummeted from 14.52% in the control to 8.59% in case of 2% UA (Figure 1F), while the food reduction rate at 2% UA was comparable (Figure S1). In response to toxin‐induced stress, genes associated with oxidative stress are often upregulated to mitigate cellular damage [9, 10, 11]. Indeed, the expression of genes involved in mitochondrial respiration (*COX4*), reactive oxygen species generation (*Duox*), and nitric oxide synthesis (*Nos*) was significantly upregulated in the larval intestine under UA stress (Figure 1G). In summary, our findings underscore the detrimental impact of UA on BSF larval development and bioconversion efficiency.

**Figure 1:**
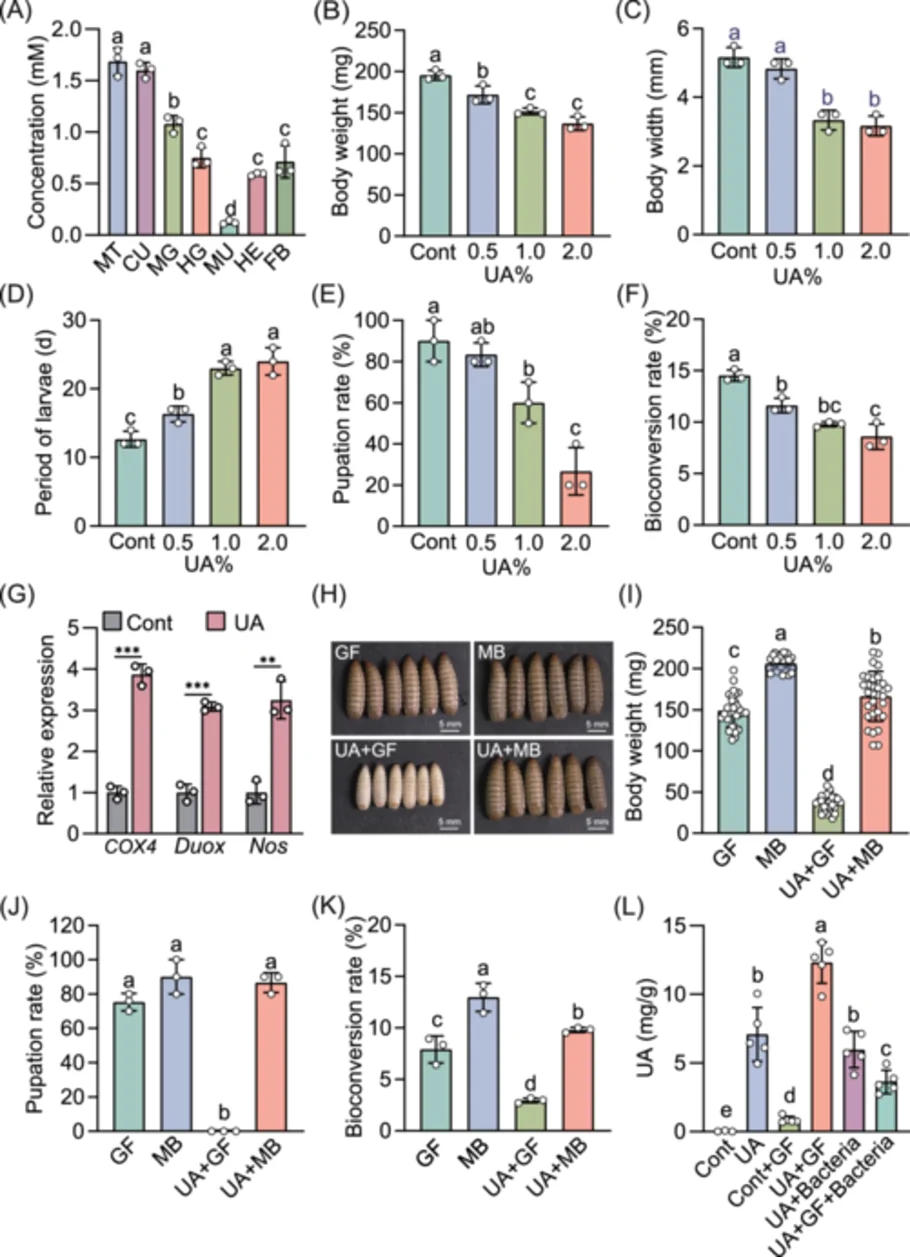
The role of the gut microbiome in uric acid detoxification in black soldier fly larvae. (A) Uric acid content in tissues of fifth‐instar larvae fed a standard diet (*n* = 3). (B–F) Effects of uric acid stress (0.5%, 1.0%, 2.0%) relative to an untreated control on BSF larval body weight, body width, developmental timing, pupation rate, and bioconversion rate (*n* = 3 independent biological replicates, with 15 larvae each). (G) The transcriptional levels of *COX4*, *Duox*, and *Nos* were assessed in larval intestines when raised on standard food with or without 1% uric acid (*n* = 3). (H) Representative images of germ‐free and mixed‐bacteria reinoculated larvae raised on standard food with or without 1% uric acid (*n* = 3 independent biological replicates, with 15 larvae each). Scale bars = 5 mm. (I–K) Effects of 1% uric acid relative to an untreated control on body weight, pupation rate, and bioconversion rate of germ‐free and mixed‐bacteria reinoculated BSF larvae (*n* = 3 independent biological replicates, with 15 larvae each). (L) Uric acid concentration in standard food with 1% uric acid after 5 days of larval treatment, microbial treatment, or co‐treatment (*n* = 5). Data are presented as mean ± standard deviation (SD). Different lowercase letters indicate significant differences between groups (*p* < 0.05), as determined by one‐way ANOVA with Tukey's HSD post hoc test in (A–F) and (I–L). Unpaired two‐tailed *t*‐test in (G); **p < 0.01; ***p < 0.001. Cont, control; CU, cuticle; FB, fat body; GF, germ‐free; HE, hemolymph; HG, hindgut; MT, Malpighian tubule; MG, midgut; MU, muscle; MB, mixed bacteria; UA, uric acid.

## 2 THE MICROBIOME MITIGATES UA TOXICITY TO LARVAL GROWTH PERFORMANCE

To study the role of the microbiome in enhancing larval tolerance to UA, germ‐free (GF) and mixed‐bacteria (MB) associated BSF larvae were generated (Figure S2A), where the axenic state was confirmed by bacterial plating (Figure S2B). Strikingly, GF larvae subjected to UA gained merely 24.6% of the body weight of their siblings under UA‐free conditions (Figure 1H,I). Moreover, MB inoculation completely restored the growth retardation observed in UA‐treated GF larvae (Figure 1H). Notably, UA‐treated MB larvae reached 80.7% of the body weight of their UA‐free siblings, suggesting that the microbiome was required to mitigate UA toxicity to larval growth (Figure 1I). In parallel, larvae challenged with UA exhibited a reduced pupation rate, which was similarly rescued by MB inoculation (Figure 1J). Our analysis also revealed that UA treatment severely reduced the bioconversion rate of GF larvae, and this adverse effect was alleviated by MB supplementation (Figure 1K).

To explore whether the microbiome contributes to UA degradation, we measured UA levels in the diet. Unanticipatedly, bacterial colonization alone led to a minor but statistically insignificant reduction in UA levels compared to the untreated control (Figure 1L). However, larvae inoculated with MB dramatically decreased UA levels in the diet (Figure 1L). Given that single GF larvae produced a high amount of UA, these findings suggest that the host synergized with the microbiome to degrade UA more efficiently. Collectively, our data suggest that the microbiome is essential for optimizing the growth of BSF larvae under UA stress.

## 3 UA ALTERS MICROBIAL COMPOSITION IN LARVAL INTESTINES

Next, we reasoned that UA treatment could alter the composition of the bacterial community. Principal coordinates analysis revealed a clear separation between the control and 1% UA‐treated groups (Figure 2A), although exposure to 1% UA affected neither microbial richness nor evenness (Figure S3A). Of note, the Permutational Multivariate Analysis of Variance (PERMANOVA) test did not reach statistical significance (*p* = 0.105), potentially due to high within‐group variation. Moreover, Enterobacteriaceae and Wohlfahrtiimonadaceae, dominant in the control group, were substantially replaced by Rhizobiaceae, Dysgonomonadaceae, and Rhodobacteraceae in the guts of UA‐treated larvae (Figure 2B). Based on PICRUSt2 predictions, the analysis revealed downregulation of enriched pathways, including metabolism of cofactors and vitamins, antimicrobial drug resistance, and protein folding, sorting, and degradation (Figure S3B). The taxonomic and functional alterations detected here provide initial evidence of UA's impact on the microbiota, but further validation with larger sample sizes is warranted to ensure generalizability.

**Figure 2:**
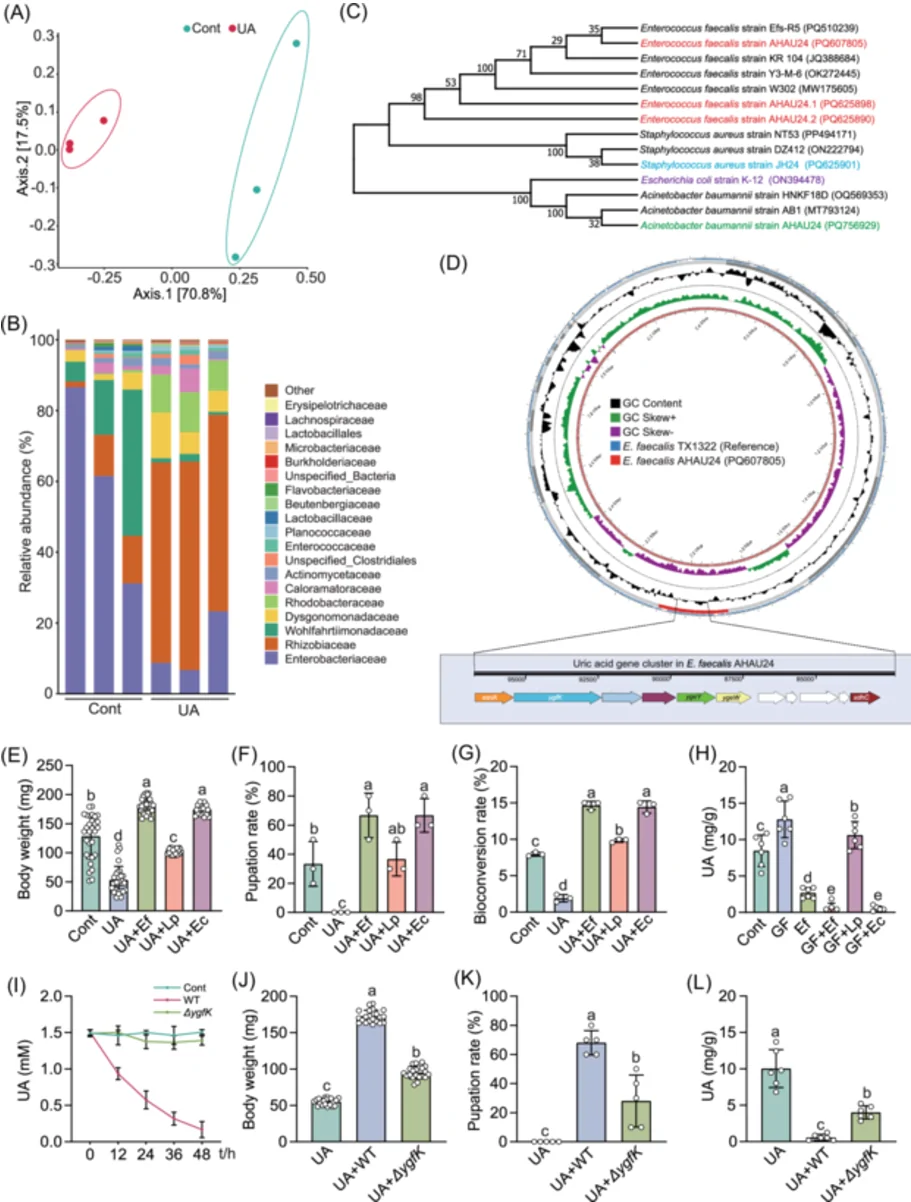
Isolation, characterization, and functional analysis of gut‐derived uric acid‐degrading bacteria. (A) Principal coordinates analysis (PCoA) based on the Bray–Curtis distances showing distinct clustering of intestinal bacteria in BSF larvae raised on standard food with or without 1% UA treatment (*n* = 3). (B) Relative abundance of the top 20 most abundant bacterial families in BSF larval intestines raised on standard food with or without 1% UA treatment (*n* = 3). (C) Phylogenetic reconstruction of UA‐degrading and related strains using the maximum likelihood method based on 16S rDNA gene sequences. (D) A CGView circular genome map of *E. faecalis* AHAU24, highlighting the genomic locations of the genes involved in the UA metabolic pathway and the UA degradation gene cluster. (E–G) Effects of 1% UA relative to an untreated control on body weight, pupation rate, and bioconversion rate of germ‐free and mono‐associated BSF larvae (*n* = 3 independent biological replicates, with 15 larvae each). (H) UA concentration in 1% UA food after 5 days of larval treatment, bacteria alone, or co‐treatment (*n* = 6). (I) Time‐course changes of UA concentrations in LB broth inoculated with wild‐type *E. coli* (WT) versus *ygfK* mutant (△*ygfK*) over 48 h (*n* = 3). (J–K) Body weight and pupation rate of GF larvae, mono‐associated with WT or Δ*ygfK* on 1% UA diet (*n* = 5 independent biological replicates, with 15 larvae each). (L) UA concentration in 1% UA food after 5‐day feeding to GF larvae, mono‐associated with WT strain or △*ygfK* (*n* = 6). Data are presented as mean ± SD. Different lowercase letters indicate significant differences between groups (*p* < 0.05), as determined by one‐way ANOVA with Tukey's post hoc test in (E–H) and (J–L). Cont, control; Ef, *E. faecalis*; Lp, *L. plantarum*; Ec, *E. coli*; GF, germ‐free; UA, uric acid.

## 4 CHARACTERIZATION OF UA‐DEGRADING BACTERIA AND GENOMIC INSIGHTS INTO UA CATABOLISM

The broad taxonomic distribution of UA‐degrading bacteria prompted us to isolate resident strains to degrade UA by using a UA‐supplemented selective medium. Five bacterial strains were isolated from BSF larval guts, including three *Enterococcus faecalis* strains, one *Staphylococcus aureus* strain, and one *Acinetobacter baumannii* strain (Figure 2C). Notably, culture‐based isolation yielded relatively less dominant taxa, likely due to the selective pressure imposed by *in vitro* UA enrichment. However, all strains we isolated exhibited the ability to grow under UA condition and degrade over 50% of total UA within 48 h of cultivation (Figure S4A), while *Lactobacillus plantarum*, a non‐UA‐degrading bacterium, failed (Figure S4A,B). Since *E. faecalis*, a member of the family Enterococcaceae ranking among the top 20 most prevalent bacterial families, has been extensively utilized as a probiotic in both humans and animals [12, 13, 14], it was chosen for further examination.

Next, we attempted to identify potential genes involved in UA degradation in *E. faecalis* AHAU24 by assembling its complete genome. The CGView genome map was generated based on the complete sequence (Figure 2D). The genome comprises a 15,881,950 bp circular chromosome with a 37.86% GC content, containing 3025 open reading frames (ORFs) predicted to encode proteins. As shown in Figure 2D, we identified a conserved set of uric‐acid‐degrading genes, including xanthine dehydrogenase (*xdhC*), hydrolase (*ygeY*), carbamoyl transferase (*ygeW*), oxidoreductase (*ygfK*), and amidohydrolase (*ssnA*), located within a 196,904 bp chromosomal fragment (Scaffold6, genomic location from 82,789 to 96,715 bp). To confirm them, we tested the expression of these genes under UA stress by the quantitative PCR technique. The result showed that UA treatment significantly increased the expression levels of these genes in *E. faecalis* (Figure S4C). Two routes for UA catabolism, converting it to either xanthine or to short‐chain fatty acids (SCFA), were identified among phylogenetically distant bacterial taxa [15]. Our results suggest that the uric‐acid‐inducible genes in *E. faecalis* AHAU24 might participate in the conversion of UA to SCFA.

## 5 *E. FAECALIS* ALLEVIATES LARVAL GROWTH ARREST UNDER UA STRESS

To further investigate whether *E. faecalis* could mitigate UA‐induced growth retardation in BSF larvae, we conducted rescue experiments by monoassociating GF larvae with *E. faecalis*, *Escherichia coli*, or *L. plantarum*. The results showed that monoassociation with the UA‐degrading bacteria, either *E. faecalis* or *E. coli*, led to an apparent increase in larval body weight (Figure 2E), whereas *L. plantarum* conferred much less benefit. Both pupation and bioconversion rates of *E. faecalis* or *E*. *coli* monoassociation larvae were also significantly higher than those of either GF or *L. plantarum*‐associated siblings (Figure 2F,G). Notably, the loads of *E. faecalis*, *E. coli*, and *L. plantarum* in larval guts were comparable after 5 days (Figure S5A). Our results furthermore showed that *E. faecalis* or *E*. *coli* monoassociation was effective in reducing the UA content in both the diet and larvae (Figure 2H and Figure S5B), underlying the alleviation of larval growth arrest under UA stress. Further genomic analysis revealed that *E. coli* K12 (GenBank accession number: ON394478) harbors a homologous uric acid gene cluster, with the key gene *ygfK* exhibiting 98% sequence identity. Due to the genetic intractability of *E. faecalis*, we turned to the more tractable *E. coli* that harbors the same uric‐acid‐inducible genes. The *ygfK* mutant of *E. coli* almost failed to degrade UA, whereas the wild‐type (WT) strain nearly consumed all UA within 48 h (Figure 2I). As anticipated, larvae associated with the *ygfK* mutant exhibited significantly lower weight gain compared to their WT‐associated counterparts (Figure 2J). Consistently, the deletion of *ygfK* significantly reduced the pupation rate in colonized larvae challenged with UA (Figure 2K). Indeed, the *ygfK* mutant was deficient in degrading UA in the diet (Figure 2L). Furthermore, we observed that WT strain supplementation mitigated the transcriptional expression of *COX4*, *Duox*, and *Nos* under UA stress, but *ygfK* mutant did not have an influence on these gene expressions compared to GF larvae (Figure S6). While our current findings provide valuable insights into the potential role of UA‐degrading genes, further investigation through cross‐species validation, gene complementation studies, or in vivo models would help strengthen the evidence for their causal involvement and enhance the generalizability of these observations.

Given that chicken manure generally contains high levels of UA, we finally investigated whether *E. faecalis* could promote the growth of larvae employed for chicken manure management. To minimize interference from resident UA‐degrading bacteria, most of the resident bacteria in the manure were killed by microwave heating for 2 min prior to inoculation with *E. faecalis*. Our data showed that an increase in body weight was observed following *E. faecalis* inoculation (Figure S7A). More importantly, the bioconversion rate of larvae was increased in group with *E. faecalis* supplementation (Figure S7B). Correspondingly, the lowest UA content was observed in chicken manure supplemented with *E. faecalis* (Figure S7C). One potential limitation of this study is that certain bacteria, including heat‐resistant or spore‐forming strains, may survive microwave heat‐killing and influence experimental outcomes. However, the large inoculum of UA‐degrading bacteria likely outcompeted any surviving microbes, so future studies should verify inactivation efficiency and consider complementary sterilization methods if complete elimination is necessary.

Here, we report that the gut microbiome is required to promote BSF development by breaking down UA. These findings agree with previous studies on insect herbivores, in which gut bacteria enable the breakdown of plant toxic compounds, such as glucosinolates in Brassicaceae or alkaloids in solanaceous plants [16]. Related UA‐degrading genes are present in the genomes of *E. faecalis* and *E. coli*, which underlie the mechanism by which these bacteria promote BSF growth performance. However, many other bacteria widely exist in the BSF gut, and additional UA‐degrading pathways need to be explored in the future. Understanding the molecular mechanisms provides insights into optimizing BSF‐rearing systems by leveraging microbial contributions to boost productivity and waste recycling efficiency [17, 18]. While aerobic Luria‐Bertani (LB)‐based approach successfully identified facultative anaerobes like *Enterococcus* as UA degraders, this method inherently selects for oxygen‐tolerant, fast‐growing bacteria, potentially overlooking anaerobic or slow‐growing taxa. Future work should adopt anaerobic culturing conditions or metagenomic analyses to capture a broader diversity of UA‐metabolizing microbes. More importantly, the potential synergism among UA‐degrading bacteria, as well as their interactions with the broader bacterial community, remains largely unexplored. Understanding these microbial interactions would offer valuable insights into how bacterial consortia collectively contribute to UA metabolism, thereby boosting the overall efficiency of BSF farming.

This study systematically investigated the effects of UA on the growth and physiological responses of BSF larvae, as well as the role of intestinal microbiota in UA degradation. Our findings demonstrate that gut bacteria play a pivotal role in mitigating UA‐induced stress in BSF larvae. Notably, *E. faecalis*, a UA‐degrading bacterium isolated from the BSF gut, confers host resistance to UA toxicity by efficiently degrading UA. Through genomic and functional analysis, we identified specific UA‐degrading genes in *E. faecalis* that are essential for alleviating UA‐related stress. Overall, these findings offer novel insights into host–microbiome interactions in response to UA toxicity, shedding light on potential microbial management strategies to mitigate UA‐induced stress.

## AUTHOR CONTRIBUTIONS

**Xiaowen Ji:** Methodology; validation; funding acquisition; investigation; writing—original draft. **Shaojie Zhou:** Methodology; validation; formal analysis; investigation; data curation. **Wenwen Chen:** Validation; formal analysis. **Bin Cao:** Validation; formal analysis. **Jingjing Zhuang:** Validation; formal analysis. **Sheng Zhang:** Validation; formal analysis. **Bingshuo Han:** Validation; formal analysis. **Benping Yang:** Validation; formal analysis. **Shuxiang Zhang:** Resources; funding acquisition. **Yanfeng Xue:** Resources. **Jibin Zhang:** Supervision; project administration. **Yinglao Zhang:** Supervision; project administration. **Wei Liu:** Supervision; project administration; funding acquisition; writing—original draft; writing—review & editing. All authors have read the final manuscript and approved it for publication.

## CONFLICT OF INTEREST STATEMENT

The authors declare no conflicts of interest.

## ETHICS STATEMENT

The animal care and all experiments were performed in accordance with the guidelines approved by the Ethics Review Committee for Animal Experimentation of Anhui Agricultural University (Permit number: KJLL2025038).

## Supporting information


**Figure S1.** The impact of uric acid on the food reduction rate.
**Figure S2.** Construction and validation of larval systems in different states.
**Figure S3.** Effects of uric acid on microbial diversity and function.
**Figure S4.** Growth and metabolic characteristics of isolated uric acid‐degrading strains.
**Figure S5.** Confirmation of gut bacterial colonization and observation of consequent changes in larval uric acid levels.
**Figure S6.** Effects of *ygfK* knockout in *E. coli* on intestinal oxidative gene expression in BSF larvae.
**Figure S7.** Effects of *E. faecalis* AHAU24 on BSF larval growth, conversion rate, and substrate uric acid content in a chicken manure rearing system.

**Table S1.** Primers for qPCR.

## Data Availability

All the raw sequencing data and the genome assembly generated for this research are available in the NCBI BioProject database under accession numbers PRJNA1203020 (https://www.ncbi.nlm.nih.gov/bioproject/?term=PRJNA1203020) and PRJNA1202998 (https://www.ncbi.nlm.nih.gov/bioproject/?term=PRJNA1202998). The data and scripts used are saved in GitHub https://github.com/jxw-123/BSF-UA. Supplementary materials (methods, figures, tables, graphical abstract, slides, videos, Chinese translated version, and update materials) may be found in the online DOI or iMeta Science http://www.imeta.science/imetaomics/.
