# Supplementary material for: The gut microbiome promotes the growth performance of black soldier fly larvae by detoxifying uric acid
Source: IMetaOmics. 2025 Dec 24;3(1):e70070. doi: 10.1002/imo2.70070 (PMC13674428; doi:10.1002/imo2.70070)
Supplement: Supplementary file 1 [file IMO2-3-e70070-s002.docx]

Supporting information to

**The gut microbiome promotes the growth performance of black soldier fly larvae by detoxifying uric acid**

**Running title**: Microbiome-mediated uric acid degradation enhances black soldier fly larval growth

Xiaowen Ji^1, 2, 3#^, Shaojie Zhou^1#^, Wenwen Chen^1#^, Bin Cao^1^, Jingjing Zhuang^1^, Sheng Zhang^1^, Bingshuo Han^1^, Benping Yang^1^, Shuxiang Zhang^2, 3^, Yanfeng Xue^4^, Jibin Zhang^2*^, Yinglao Zhang^3*^, Wei Liu^1*^

^1^Anhui Province Key Laboratory of Crop Integrated Pest Management, School of Plant Protection, Anhui Agricultural University, Hefei, 230036, China

^2^National Key Laboratory of Agricultural Microbiology, College of Life Science and Technology, Huazhong Agricultural University, Wuhan, 430070, China

^3^Anhui Province Key Laboratory of Resource Insect Biology and Innovative Utilization, School of Life Sciences, Anhui Agricultural University, Hefei, 230036, China

^4^College of Animal Science and Technology, Anhui Agricultural University, Hefei, 230036, China

^#^These authors contributed equally: Xiaowen Ji, Shaojie Zhou, Wenwen Chen.

^*^Correspondence: [zhangjb@mail.hzau.edu.cn](mailto:zhangjb@mail.hzau.edu.cn) (Jibin Zhang); zhangyl@ahau.edu.cn (Yinglao Zhang); liuwei5@ahau.edu.cn (Wei Liu)

**METHODS**

**Black soldier fly strain**

The black soldier fly (BSF), *Hermetia illucens*, used in this study was obtained from the State Key Laboratory of Agricultural Microbiology at Huazhong Agricultural University, China. BSF larvae were reared using a previously described method with modifications [1]. Briefly, the flies of all treatment groups were reared in glass jars under conditions: 28°C, 65% relative humidity, and a 14:10 light/dark photoperiod. Illumination was provided by daylight white fluorescent tubes with an irradiance of approximately 20 W/m². Larvae were fed an artificial diet consisting of wheat bran mixed with water to achieve a final moisture content of 66.7%. Adult flies were supplied with 5% sucrose solution, and egg-laying was stimulated with iodine-tungsten lamps.

**Bacterial isolation and identification**

To isolate bacteria from the gut, fifth instar larvae were surface-sterilized twice by immersion in 75% ethanol for 1 min each, and then rinsed twice in sterile phosphate buffered saline (PBS) at pH 7.4. The total gut, consisting of pools of 100 larvae, was aseptically dissected, transferred to 10 mL of sterile PBS, and homogenized. UA-degrading bacteria were first enriched in LB broth supplemented with 0.025% (w/v) UA, a concentration determined through solubility testing. For colony isolation, diluted cell suspensions were plated on Luria-Bertani (LB) agar and incubated at 37°C for 24 h. All morphologically distinct bacterial species were subcultured three times to ensure their purity and stability. All 16S rDNA sequence data of isolated strains are publicly available on the NCBI database, including *Enterococcus faecalis* (GenBank accession number: PQ625898; PQ607805; PQ625890), *Staphylococcus aureus* (GenBank accession number: PQ625901), and *Acinetobacter baumannii* (GenBank accession number: PQ756929).

**Bacterial strains and culture conditions**

Single colonies of isolated strains, *Escherichia coli* K12 (GenBank accession number: ON394478), and *Lactobacillus plantarum* (GenBank accession number: KY038178) were cultured in LB broth and incubated at 37°C with continuous shaking. After overnight incubation, the bacterial culture was centrifuged, and the pellet was resuspended in 1 mL PBS for inoculation (OD_600_ = 1).

**Construction of the *ygfK*‑deficient mutant**

Homologous recombination was employed to delete *ygfK* from the chromosome of *E*. *coli* K12 (GenBank accession number: ON394478) using the lambda Red recombinase system [2, 3]. In brief, the pKD3 plasmid served as a template to amplify a fragment containing the chloramphenicol-resistance cassette inserted within the *ygfK* gene, using primers K12-*ygfK*-F and K12-*ygfK*-R. This fragment was transformed into K12 competent cells with lambda Red recombinase from the pKD46 plasmid via electroporation. Mutants were selected with LB plates containing 15 μg/mL chloramphenicol. The *ygfK* mutant was verified with PCR amplification using the primers check-*ygfK*-F and check-*ygfK*-R. The plasmid pCP20 was subsequently transformed into the mutant strain to remove the chloramphenicol cassette, confirming by PCR amplification with the primers check-*ygfK*-F and check-*ygfK*-R. The PCR products were further validated by DNA sequencing, and the mutant strain was designated as Δ*ygfK*. The primers used were listed in Table S1.

**Uric acid (UA) treatment for bacteria and larvae**

Bacteria were treated with LB broth supplemented with or without 0.025% (w/v) UA, and bacterial growth was monitored by measuring OD_600_ at 12-hour intervals for 48 h using a UV-visible spectrophotometer. Meanwhile, UA concentration in the medium was measured at 510 nm with a Thermo Multiskan FC microplate reader according to the UA kit protocol (Nanjing Jiancheng, Catalog No.: C012-2-1).

For the UA treatment for conventionally-reared BSF larvae, 5-day-old larvae were randomly assigned to glass bottles, with each group constituting three biological replicates (each containing 15 larvae). The bottle contained an artificial diet of 5 g wheat bran reconstituted with either 10 mL tap water (control) or UA solutions at final concentrations of 0.5%, 1.0%, or 2.0% (w/w), which were chosen based on the range found in poultry manure [4, 5]. Following a 5-day exposure period, larvae were harvested for growth performance assessment.

**Bacteria draftmap method**

The genomic analysis of *E. faecalis* AHAU24 was performed using Illumina sequencing platforms at Novogene Bioinformatics Technology Co., Ltd (Beijing, China), which generated high-quality short-read data. Library construction involved random fragmentation of genomic DNA, adapter ligation, size selection, and PCR amplification, followed by quantification using Qubit and bioanalyzer. Sequencing was conducted with Illumina instruments to achieve sufficient coverage for downstream assembly. For genome assembly, multiple software tools were employed to optimize accuracy: SOAPdenovo (v2.04) [6] with default K-mers (95, 107, 119), SPAdes (v3.13.0) [7] using K-mers 99 and 127, and ABySS (v2.1.5) [8] with K-mer 64. The assemblies were integrated using CISA to select the scaffold with the fewest contigs, followed by gap filling with GapCloser. Low-depth reads (< 0.35× average depth) and fragments < 500 bp were filtered to yield the final assembly. Genome quality metrics included N50, contig number, and total assembly size, with validation based on clean data after stringent filtering (Q20 > 80%, adapter removal, and host contamination checks). Annotation utilized GeneMarkS [9] for coding sequences. Functional prediction involved BLAST against Gene Ontology (GO) [10], Kyoto Encyclopedia of Genes and Genomes (KEGG) [11], Clusters of Orthologous Groups (COG) [12], NCBI non-redundant (NR) [13], and Swiss-Prot databases (E-value < 1e-5, alignment length > 40%) [14]. The draft genome sequence of *E. faecalis* was deposited in GenBank under the accession number BioProject PRJNA1202998.

**BSF growth performance assay**

After a 5-day UA treatment, each larva was collected for weight measurement with an analytical balance (Sartorius BSA124S). The larvae were photographed using a Nikon stereomicroscope, and the body width was subsequently measured using ImageJ software. To assess the pupation rate, larvae were transferred to new containers upon reaching the prepupal stage, and the pupation rate was recorded accordingly.

**Generation of germ-free and bacteria-reintroduced larvae**

Embryos were collected within 24 h of egg-laying, rinsed with tap water, and transferred to Eppendorf tubes. A series of sterilization treatments was applied, including exposure to diluted Walch sanitiser (1:30, Whealthfields Lohmann, Germany), 2.5% sodium hypochlorite (SH; Sigma-Aldrich, St. Louis, MO, USA), 75% ethanol, and sterile PBS containing 0.01% Triton X-100. The sterilized embryos were then transferred to vials containing autoclaved food for breeding until the larvae reached the same size as the 5-day-old conventionally-reared group. The food recipe for GF larvae breeding was as follows: 105 g dextrose, 7.5 g agar, 26 g yeast, 50 g cornmeal, and 1 L purified water were mixed, boiled for 30 min with constant agitation, and autoclaved. To confirm the axenic status of BSF larvae, homogenates were plated on nutrient agar plates prepared with peptone (10 g/L), beef extract powder (3 g/L), NaCl (5 g/L), and agar. To generate bacteria-reintroduced BSF larvae, either a specific strain or mixed bacteria (MB) was inoculated to axenic wheat bran food (66.7% moisture content) vials containing germ-free (GF) larvae, with or without 1% UA. The “MB” consortium came from the gut microbiota of BSF larvae. Specifically, we dissected 100 conventionally-reared larval guts, homogenized them in 10 mL sterile PBS supplemented with 15% glycerol, and then aliquoted and stored the mixture at –20°C. For inoculation, 0.1 mL of this homogenate was thawed and added directly to the sterile wheat bran diet described above.

**Quantification of bacterial colonization in black soldier fly larval gut**

To assess the colonization of a single bacterial strain in the BSF gut, dissected guts were homogenized in sterile PBS. Serial 10-fold dilutions of the homogenates were plated on LB agar, and after overnight incubation at 37°C, CFU per gut were counted from plates containing 30–300 discrete colonies.

**Quantification of UA content in larvae and diet**

To examine UA levels in specific tissues of fifth-instar larvae, various body parts were carefully dissected in cold phosphate-buffered saline (PBS, pH 7.4) and homogenized. For UA measurement in the larval body, intact larvae were homogenized directly in cold PBS. The absorbance of UA was measured at 510 nm using a Thermo Multiskan FC microplate reader according to the UA kit protocol (Nanjing Jiancheng, Catalog No.: C012-2-1). Relative UA levels were normalized to protein content, which was quantified using the BCA Protein Quantification kit (Vazyme, Catalog No.: E112-01) following the manufacturer’s instructions. To determine the UA content in the diet, 1 g of food was diluted with sterile water, and the supernatant was assayed under the same conditions as the tissue samples. All measurements were performed in triplicate to ensure reproducibility.

**Diet reduction rate and bioconversion rate calculation**

After 5-day UA treatment, 10-day-old BSF larvae were collected. Next, BSF larvae and wheat bran diet were dried in an oven at 55°C for 16 h. The reduction rate and conversion rate of the wheat bran were determined as follows:

$$\text{Reduction rate }\left( \text{\%} \right)\text{=}\frac{\text{S}\text{1-S2}}{\text{S}\text{1}}\text{×100}$$

$$\text{C}\text{onversion rate }\left( \text{\%} \right)\text{=}\frac{\text{D}\text{1-D2}}{\text{S}\text{1-S2}}\text{×100}$$

D1 represents the dry weight of BSF larvae after rearing on the diet, while D2 represents the initial dry weight of BSF larvae of the same developmental stage and size prior to diet treatment. Similarly, S1 and S2 denote the dry weights of the diet before and after rearing, respectively.

**16S rDNA gene sequencing of the microbiome**

Fifteen fifth-instar larvae were selected from glass-rearing jars. The surface was sequentially disinfected with 75% ethanol and washed with sterile water. Guts were dissected and ground in cold sterile PBS. Homogenates were centrifuged at 1000 rpm for 3 min to remove host tissue in the precipitate. The supernatant was centrifuged at 6000 rpm for 1 min to collect the bacterial pellets, which were then frozen in liquid nitrogen. All samples were sequenced at Novogene Bioinformatics Technology Co., Ltd (Beijing, China). To amplify the 16S rDNA V4 and V5 regions of the bacteria, the primers 515F (5′-GTGCCAGCMGCCGCGGTAA-3′) and 907R (5′-CCGTCAATTCCTTTGAGTTT-3′) were used. The PCR products with proper size were selected by 2% agarose gel electrophoresis. Same amount of PCR products from each sample was pooled, end-repaired, A-tailed, and further ligated with Illumina adapters. Libraries were sequenced on a paired-end Illumina platform to generate 250 bp paired-end raw reads. Subsequently, library quality was assessed and quantified by qPCR. Quantified libraries were pooled and sequenced on Illumina platforms, according to effective library concentration. Sufficient depth (over 100,000 reads per sample) was achieved for robust microbial community analysis. Rarefaction analysis was performed to confirm adequate community coverage. After high-throughput sequencing and bioinformatics analysis, amplicon sequence variants (ASVs) were obtained using the DADA2 package. Raw reads were filtered by truncating forward/reverse reads at 240/200 bp (truncLen) and excluding reads with expected errors > 2 (maxEE). All statistical analyses were conducted using R version 4.3.2 for Windows. The ASV table and taxonomic assignments were imported and assembled into a phyloseq object for downstream analysis. Alpha diversity indices (Chao1 and Shannon indices) were calculated and statistically compared across groups using the phyloseq and vegan packages. To analyze beta diversity, we performed Principal Coordinates Analysis (PCoA) based on Bray-Curtis distance (for taxonomic composition). Predictive functional profiling of bacterial communities at KEGG pathway level 3 was inferred using PICRUSt2 (version 2.5.2) based on the KEGG database; the resulting pathway abundance table was imported into R (using phyloseq) for subsequent statistical analysis and visualization. PERMANOVA (999 permutations) was performed on Bray-Curtis dissimilarities using the vegan package. Raw 16S rDNA gene amplicon sequence data derived from UA stress experiments are publicly available under NCBI BioProject number PRJNA1203020.

**Chicken manure experiment**

The chicken manure was obtained from Feichi Poultry Ecological Breeding Co., Ltd. (Hefei, China). For the experimental setup, 5-day-old BSF larvae (*n* = 3 independent biological replicates, with 10 larvae each) were transferred into glass bottles each containing 30 g of either untreated (control) or oven-pretreated (heat, 2 min) chicken manure. After 5 days of treatment, larval growth performance, bioconversion rate, and UA concentration in chicken manure were measured as described above.

**RNA extraction and quantitative PCR**

Total RNA was isolated from each group using five larval guts per biological replicate (with three biological replicates per group), using the TRIzol reagent (Vazyme Catalog No.: R403-1) according to the manufacturer’s instructions. The RNA was eluted with 20 μL of DNase/RNase-free water by centrifugation at 12,000 g for 30 s. RNA concentration was determined using a NanoDrop 1000 Spectrophotometer (Thermo Scientific). cDNA was synthesized from 1 μg of total RNA using HiScript III All-in-one RT SuperMix (Vazyme Catalog No.: R333-01). qPCR was performed in 20 μL reactions containing 10 μL SYBR Mix (Vazyme Catalog No.: Q711-02), 1 μL cDNA template, and 0.25 μM of each gene-specific primer. Amplification was monitored using a Bio-Rad CFX Connect Optics Module. The *rp49* gene served as the reference gene for samples.

For analysis of the expressions of UA-degrading genes in *E. faecalis* AHAU24, bacteria were cultured in defined medium with or without 0.025% (w/v) UA at 37°C for 24 h (three biological replicates per group). RNA was isolated from bacterial cells of 10 mL culture per replicate. cDNA synthesis and qPCR followed the same protocol as above, with 16S rRNA as the reference gene. Primer sequences are listed in Table S1.

**Statistical analysis**

Data were analyzed using GraphPad Prism 8.4.3 (La Jolla, CA, USA) software. Comparisons between two samples were made by unpaired two-tailed *t*-test. Comparisons among multiple samples were made by one-way ANOVA with Tukey’s HSD post-hoc test. Normality and variance homogeneity assumptions were verified prior to inter-group comparisons. *p* values < 0.05 were considered statistically significant. All statistical analyses and definitions of sample sizes are provided in the figure legends. At least three biological replicates were tested unless otherwise specified. Error bars in bar graphs or line graphs represent the standard deviation (SD) of the data.

**REFERENCES**

1. Li, Xinyu, Cheng Mei, Xingyu Luo, Dilinuer Wulamu, Shuai Zhan, Yongping Huang, Hong Yang. 2023. “Dynamics of the intestinal bacterial community in black soldier fly larval guts and its influence on insect growth and development.” *Insect Science* 30: 947-963. <https://doi.org/10.1111/1744-7917.13095>

2. Datsenko, Kirill A., Barry L. Wanner. 2000. “One-step inactivation of chromosomal genes in *Escherichia coli* K-12 using PCR products.” *Proceedings of the National Academy of Sciences of the United States of America* 97: 6640-6645. <https://doi.org/10.1073/pnas.120163297>

3. Wang, Hui, Fei Shang, Jiawei Shen, Jingyi Xu, Xiaolin Chen, Jingtian Ni, Lumin Yu, Ting Xue. 2021. “LsrR, the effector of AI-2 quorum sensing, is vital for the H_2_O_2_ stress response in mammary pathogenic *Escherichia coli*.” *Veterinary Research* 52: 127. <https://doi.org/10.1186/s13567-021-00998-8>

4. Selle, Peter H., David I. Cantor, Leon R. McQuade, Bernard V. McInerney, Juliano Cesar de Paula Dorigam, Shemil P. Macelline, Peter V. Chrystal, Sonia Y. Liu. 2021. “Implications of excreta uric acid concentrations in broilers offered reduced crude protein diets and dietary glycine requirements for uric acid synthesis.” *Animal Nutrition* 7: 939-946. <https://doi.org/10.1016/j.aninu.2021.03.011>

5. Keiichi, Murakami, Hara Masayuki, Kondo Takuya, Hashimoto Yohey. 2011. “Increased total nitrogen content of poultry manure by decreasing water content through composting processes.” *Soil Science and Plant Nutrition* 57: 705-709. <https://doi.org/10.1080/00380768.2011.616856>

6. Li, Ruiqiang, Hongmei Zhu, Jue Ruan, Wubin Qian, Xiaodong Fang, Zhongbin Shi, Yingrui Li, et al. 2010. “De novo assembly of human genomes with massively parallel short read sequencing.” *Genome Research* 20: 265-272. <https://doi.org/10.1101/gr.097261.109>

7. Bankevich, Anton, Sergey Nurk, Dmitry Antipov, Alexey A. Gurevich, Mikhail Dvorkin, Alexander S. Kulikov, Valery M. Lesin, et al. 2012. “SPAdes: A New Genome Assembly Algorithm and Its Applications to Single-Cell Sequencing.” *Journal of Computational Biology* 19: 455-477. <https://doi.org/10.1089/cmb.2012.0021>

8. Simpson, Jared T, Kim Wong, Shaun D Jackman, Jacqueline E Schein, Steven J M Jones, Inanc Birol. 2009. “ABySS: A parallel assembler for short read sequence data.” *Genome Research* 19: 1117-1123. <https://doi.org/10.1101/gr.089532.108>

9. Besemer, John, Alexandre Lomsadze, Mark Borodovsky. 2001. “GeneMarkS: a self-training method for prediction of gene starts in microbial genomes. Implications for finding sequence motifs in regulatory regions.” *Nucleic Acids Research* 29: 2607-2618. <https://doi.org/10.1093/nar/29.12.2607>

10. Ashburner, Michael, Catherine A. Ball, Judith A. Blake, David Botstein, Heather Butler, J. Michael Cherry, Allan P. Davis, et al. 2000. “Gene Ontology: tool for the unification of biology.” *Nature Genetics* 25: 25-29. <https://doi.org/10.1038/75556>

11. Kanehisa, Minoru, Susumu Goto, Shuichi Kawashima, Yasushi Okuno, Masahiro Hattori. 2004. “The KEGG resource for deciphering the genome.” *Nucleic Acids Research* 32: D277-D280. <https://doi.org/10.1093/nar/gkh063>

12. Galperin, Michael Y., Kira S. Makarova, Yuri I. Wolf, Eugene V. Koonin. 2015. “Expanded microbial genome coverage and improved protein family annotation in the COG database.” *Nucleic Acids Research* 43: D261-D269. <https://doi.org/10.1093/nar/gku1223>

13. Li, Weizhong, Lukasz Jaroszewski, Adam Godzik. 2002. “Tolerating some redundancy significantly speeds up clustering of large protein databases.” *Bioinformatics* 18: 77-82. <https://doi.org/10.1093/bioinformatics/18.1.77>

14. Bairoch, Amos, Rolf Apweiler. 1999. “The SWISS-PROT protein sequence data bank and its supplement TrEMBL in 1999.” *Nucleic Acids Research* 27: 49-54. <https://doi.org/10.1093/nar/27.1.49>

**Figure S1 The impact of uric acid on the food reduction rate.** Effects of uric acid stress (0.5%, 1.0%, 2.0%) relative to an untreated control on the reduction rate of wheat bran (*n* = 3). Data represent mean ± SD. Significant differences were determined by one-way ANOVA with Tukey’s HSD post-hoc test. Cont, control; UA, uric acid.

**Figure S2 Construction and validation of larval systems in different states.** (A) Schematic illustration of generating germ-free larvae and larvae supplemented with mixed bacteria. Embryos were sterilized successively using sanitiser Walch, sodium hypochlorite, ethanol, and PBS containing 0.01% Triton X-100 (PBST). BSF indigenous bacteria were inoculated to generate mixed-bacteria larvae. (B) Sterility validation of BSF larvae after 5-day uric acid treatment via homogenization and plate culturing. GF, germ free; MB, mixed bacteria; SH, sodium hypochlorite.

**Figure S3 Effects of uric acid on microbial diversity and function.** (A) Effects of uric acid stress on bacterial diversity (Chao1 and Shannon indices) in BSF larval intestines after 5-day exposure to 1% uric acid. Data are presented as mean ± SD (*n* = 3). *p*-values were calculated using two-tailed unpaired Student’s *t*-tests. (B) Cluster analysis of relative abundance based on PICRUSt2 functional annotation of intestinal bacteria from the two treatments. The top 12 KEGG pathways showing statistically significant differences (*p* < 0.05) are displayed. Cont, control; UA, uric acid.

**Figure S4 Growth and metabolic characteristics of isolated uric acid-degrading strains.** (A) Changes in uric acid concentration during bacteria growth (*E. faecalis*, *L. plantarum*, *A. baumannii*, and *S. aureus*) in LB medium with uric acid addition (*n* = 6). (B) Growth (OD_600_) of *E. faecalis* and *L. plantarum* cultured in LB medium with or without 0.025% uric acid (*n* = 3). (C) Transcriptional levels of *ssnA*, *ygfK*, *ygeY*, *ygeW*, and *xdhC* in *E. faecalis* cultured in LB medium with or without 0.025% uric acid (*n* = 3). Data are presented as mean ± SD. *p*-values were calculated using two-tailed unpaired Student’s *t*-tests. ****p* < 0.001. Cont, control; UA, uric acid; Ef, *E. faecalis*; Lp, *L. plantarum*; Ab, *A. baumannii*; Sa, *S. aureus*.

**Figure S5** **Confirmation of gut bacterial colonization and observation of consequent changes in larval uric acid levels.** (A) Colony-forming unit levels of bacteria in black soldier fly larval gut after 5-day exposure to 1% uric acid (*n* = 3). (B) Effect of bacteria colonization on uric acid levels in BSF larval bodies (*n* = 5). Data are presented as mean ± SD. Different lowercase letters indicate significant differences between groups (*p* < 0.05), as determined by one-way ANOVA with Tukey’s HSD post-hoc test. Cont, control; UA, uric acid; Ef, *E. faecalis*; Lp, *L. plantarum*; Ec, *E. coli*; CFU, colony-forming unit.

**Figure S6 Effects of *ygfK* knockout in *E. coli* on intestinal oxidative gene expression in BSF larvae.** Oxidative stress gene expression (*COX4*, *Duox*, and *Nos*) in the intestine of germ-free BSF larvae or those monoassociated with *E. coli* K12 wild type, Δ*ygfK* under 1% uric acid stress (*n* = 3). Data are presented as mean ± SD. Different lowercase letters indicate significant differences between groups (*p* < 0.05), as determined by one-way ANOVA with Tukey’s HSD post-hoc test. Cont, control; UA, uric acid; WT, wild type.

**Figure S7 Effects of *E. faecalis* AHAU24 on BSF larval growth, conversion rate, and substrate uric acid content in a chicken manure rearing system.** (A and B) Body weight and bioconversion rate of larvae raised on conventional or heat-pretreated chicken manure, with or without *E. faecalis* AHAU24 inoculation (*n* = 3 independent biological replicates, with 10 larvae each). (C) Uric acid concentrations in chicken manure after 5 days of larval treatment with *E. faecalis* AHAU24 inoculation, compared to untreated controls (*n* = 3). Data are presented as mean ± SD. Different lowercase letters indicate significant differences between groups (*p* < 0.05), as determined by one-way ANOVA with Tukey’s HSD post-hoc test. Cont, control; Ef, *E. faecalis*.
